# Modeling the molecular fingerprint of protein-lipid interactions of MLKL on complex bilayers

**DOI:** 10.3389/fchem.2022.1088058

**Published:** 2023-01-12

**Authors:** Ricardo X. Ramirez, Oluwatoyin Campbell, Apoorva J. Pradhan, G. Ekin Atilla-Gokcumen, Viviana Monje-Galvan

**Affiliations:** ^1^ Department of Chemical and Biological Engineering, School of Engineering and Applied Sciences, University at Buffalo, Buffalo, NY, United States; ^2^ Department of Chemistry, College of Arts and Sciences, University at Buffalo, Buffalo, NY, United States

**Keywords:** protein-lipid interactions, lipid membrane modeling, local lipid fingerprint, MLKL protein, molecular dynamics simulations, mechanisms of cell death

## Abstract

Lipids, the structural part of membranes, play important roles in biological functions. However, our understanding of their implication in key cellular processes such as cell division and protein-lipid interaction is just emerging. This is the case for molecular interactions in mechanisms of cell death, where the role of lipids for protein localization and subsequent membrane permeabilization is key. For example, during the last stage of necroptosis, the mixed lineage kinase domain-like (MLKL) protein translocates and, eventually, permeabilizes the plasma membrane (PM). This process results in the leakage of cellular content, inducing an inflammatory response in the microenvironment that is conducive to oncogenesis and metastasis, among other pathologies that exhibit inflammatory activity. This work presents insights from long all-atom molecular dynamics (MD) simulations of complex membrane models for the PM of mammalian cells with an MLKL protein monomer. Our results show that the binding of the protein is initially driven by the electrostatic interactions of positively charged residues. The protein bound conformation modulates lipid recruitment to the binding site, which changes the local lipid environment recruiting PIP lipids and cholesterol, generating a unique fingerprint. These results increase our knowledge of protein-lipid interactions at the membrane interface in the context of molecular mechanisms of the necroptotic pathway, currently under investigation as a potential treatment target in cancer and inflamatory diseases.

## Introduction

The plasma membrane (PM) is the natural barrier that encapsulates cells and cellular organelles; it is composed primarily of lipids arranged in a bilayer, proteins, and sugars (Corradi et al., 2018). Lipids constitute the structural backbone of the membrane, and their relative composition modulates membrane tension, rigidity, and shape (Casares and EscribRossello, 2019). Furthermore, lipids have a dynamic interaction with transmembrane and peripheral membrane proteins (Kandt et al., 2008; Sapay and Tieleman, 2008; Monje-Galvan and Klauda, 2018) that is relevant to cell signaling cascades, ionic flux, cargo transport, mechanisms of cell death, and disease progression. The molecular-level understanding of these protein-lipid interactions at the membrane interface is relevant to understand mechanisms of membrane permeabilization and cell death. This knowledge can be potentially leveraged in the treatment of several diseases such as cancer.

For instance, necroptosis is a caspase-independent programmed cell death pathway under consideration as a potential cancer treatment (Wang et al., 2017; Gong et al., 2019; Qin et al., 2019). This pathway initiates when the tumor necrosis factor TNF-α binds its receptor and ends with the permeabilization of the PM and the leakage of cellular content. The process responsible for PM permeabilization is the interaction of mixed lineage kinase-like (MLKL) protein with membrane lipids. MLKL is the final executor of necroptosis by translocating to the PM and causing membrane disruption (Galluzzi et al., 2017; Chen et al., 2019; Choi et al., 2019). However, the details of these protein-lipid interactions and the corresponding membrane permeabilization mechanism are unknown. Necroptosis is a relevant pathway in cancer, and also in neurodegenerative and inflammatory diseases (Choi et al., 2019). Similarly, there are other diseases where protein-lipid interactions alter normal function, such as in disruption of lipid metabolism in hepatitis C (Lee et al., 2020a); hence, there is an urgency to characterize their molecular mechanisms and understand the role of specific protein-lipid interactions in membrane remodeling as well as their relevance in the overall disease onset and progression.

MLKL is a pseudo-kinase with 469 residues distributed into three domains: the four helical bundle (4HB), residues 1-121; the brace, residues 133-175; and the pseudo-kinase domain (PsK), residues 193-459 (Zhang et al., 2016; Murphy, 2020; Petrie et al., 2020; Sethi et al., 2022a). MLKL is phosphorylated in preparation for the last step of necroptosis, currently considered a critical step in MLKL protein oligomerization. The 4HB domain of the oligomerized MLKL translocates to the PM and permeabilizes it; studies on MLKL lacking this domain show increase in cell viability (Zhang et al., 2021). The brace region consists of two helices and affects the interaction of the 4HB with the PM. Once the interaction between the brace and 4HB is disrupted (i.e., salt bridge between R30 and E136 breaks down), the 4HB interacts with and inserts in the PM (Su et al., 2014). Furthermore, decreasing the membrane binding of MLKL by inhibiting its S-acylation increases cell viability and restores membrane integrity (Parisi et al., 2019; Pradhan et al., 2021).

There is not yet a consensus on how the oligomerized MLKL permeabilizes the membrane. Some authors believe that it penetrates the membrane forming ion channels where cell content can leak (Zhang et al., 2021). However, other authors claim that, instead of forming ion channels, the 4HB forms cation channels or pores that allow cell content to flow (Xia et al., 2016). Furthermore, two additional models propose alternative mechanisms for membrane permeabilization, the carpet model and the toroidal pore model (Grage et al., 2016; Engelberg and Landau, 2020; Flores-Romero et al., 2020). Interestingly, the carpet model does not require the protein to cross the membrane. To increase our understanding of protein-lipid interactions in the context of mechanisms of cell death, we present an initial molecular dynamics study of a single MLKL protein with a complex lipid membrane model that mimics the environment of the PM. Our results suggest that binding of MLKL modulates lipid recruitment and can generate a unique lipid fingerprint enriched in phosphatidylinositol phosphates and cholesterol lipids at the protein binding site. These changes also affect the packing of lipids on the membrane surface of the binding leaflet, further modulating membrane surface topology and charge distribution. Proposing a final mechanism of membrane permeabilization is out of the scope of this work, which is intended as the first step in subsequent computational studies to characterize protein-lipid interactions in the context of MLKL-driven membrane remodeling and disruption.

## Methods

### Simulations setup

We used all-atom molecular dynamics simulations to model the interaction between a single MLKL protein (PDBID: 4BTF) and the PM as a starting point to characterize the molecular driving forces of late-stage necroptosis. The protein sequence corresponds to a murine model for MLKL, selected because its complete sequence of joint protein domains was available on the PDB server; on the contrary, the human MLKL tertiary structure is only available for separate domains on the PDB. Supplementary Figure S1 shows the sequence alignment for the N-terminus of the protein, namely the 4HB and Brace domains, between the human (Uniprot: Q8NB16) and murine (PDBID: 4BTF) models showing excellent agreement between the structures.

The membrane model was based on the HT-29 cell line, built with a mixture of dioleoyl-phosphatidylcholine (DOPC): cholesterol (Chol): dioleoyl-phosphatidylethanolamine (DOPE): palmitoyl-oleoyl-phosphatidylinositol-4-phosphate (POPI-1,4): palmitoyl-oleoyl-phosphatidylinositol-(2,5)-bisphosphate (POPI-2,5) (40:32:20:4:4 mol%) to model the PM; hereon after, POPI-1,4 and POPI-2,5 are referred to as PIP and PIP_2_. The membrane model was built using CHARMM-GUI *Membrane Builder* (Jo et al., 2007; Go and Jones, 2008; Jo et al., 2009; Cai et al., 2014; Lee et al., 2019), and the protein was solvated in a three-site water model using the *Solution Builder* (Lee et al., 2016; Lee et al., 2020b). The membrane model was built with 600 lipids per leaflet, fully hydrated with at least 50 water molecules per lipid. The default step-wise relaxation protocol from CHARMM-GUI was used for initial minimization and equilibration of the protein and membrane systems separately. Membrane-only systems were equilibrated for 200 ns, while the protein-water system was equilibrated for 50 ns before merging the equilibrated coordinates.

Upon equilibration, membrane and protein coordinates were merged and the simulation box rendered neutral using .15 mM KCl. The protein was positioned at different orientations above the lipid bilayer to ensure unbiased binding: (Rep1) vertical, with the pseudo-kinase domain facing membrane; (Rep2) vertical, with 4HB facing membrane; (Rep3) horizontal, with the brace facing away from the membrane; and (Rep4) horizontal, with the brace facing towards the membrane. Supplementary Figure S2 illustrates these orientations relative to the membrane surface. Table 1 summarizes the details of each system built for this study. The systems built for Rep1 and Rep2 are larger than Rep3 and Rep4 in terms of number of atoms and the *z* box vector. Rep1 and Rep2 started with the protein from a vertical conformation and had more water molecules to prevent the protein from interacting with image atoms from the bilayer during the simulation. Rep3 and Rep4 started with a horizontal protein, and were built in a smaller box to reduce the number of water molecules and reduce the computational cost. All systems were run with periodic boundary conditions and monitored to ensure no central atoms were interacting with its image atoms or with both membrane leaflets at the same time due to periodicity. The protein-membrane systems were run for 100ns to ensure they were equilibrated prior to transferring them to the Anton2 machine. The four replicas were run on this resource for at least 2,000 ns each, for a total of 8.76 μs of simulated trajectory.

**TABLE 1:** Protein-membrane simulation systems.

System	# Water molecules	Total # atoms	Box cell size (*x, y, z,* in nm)	Sim. Time (ns)
Rep1	173,744	669,706	17.1 × 17.1 × 22.3	2,180
Rep2	173,750	669,724	17.2 × 17.2 × 21.9	2,180
Rep3	138,227	562,959	17.3 × 17.3 × 18.4	2,200
Rep4	135,548	554,910	17.3 × 17.3 × 18.2	2,200

All systems were run using the CHARMM36m force field (Klauda et al., 2010; Vanommeslaeghe and MacKerell, 2012; Huang et al., 2017) and periodic boundary conditions. The Initial equilibration for the membrane-only and protein-only systems were performed using GROMACS (Abraham et al., 2015) with a timestep of 2 fs. Temperature was kept constant at 310.15 K using the Berendsen thermostat with a 1.0 ps coupling constant (Berendsen et al., 1984). Pressure was set at 1 bar and controlled semi-isotropically with the Berendsen barostat using a coupling time of 5.0 ps and compressibility of 4.5 × 10^−5^ (Berendsen et al., 1984). The merged protein-membrane systems were run with NPT dynamics, using the Nose-Hoover thermostat (Nosé, 1984; Hoover, 1985) and Parrinello-Rahman barostat (Parrinello and Rahman, 1981; Nosé and Klein, 1983); coupling and compressibility settings were kept as listed above. Non-bonded interactions were modeled using Verlet force-switch function with cutoffs set at 1.0 and 1.2 nm for Lennard-Jones interactions (Grubmüller et al., 1991). Particle Mesh Ewald was used for long-range electrostatics (Darden et al., 1993), and the LINCS algorithm (Hess et al., 1997) to constrain bonds with hydrogen atoms. The equilibration trajectories were run with resources available at the Center for Computational Research (CCR) at the University at Buffalo (Center for Computational Research UaB, 2019).

The production runs for each protein-membrane replica were computed on the Anton2 machine (Shaw et al., 2014a; Shaw et al., 2014b), hosted at the Pittsburgh Supercomputing Center (PSC). Simulation parameters were set by Anton2 internal guesser files, which are automated scripts designed to optimize the parameters for the integration algorithms of this machine. As such, the cut-off values to compute non-bonded interactions between neighboring atoms were set automatically during system preparation. Long-range electrostatics were computed using the Gaussian Split Ewald algorithm (Shan et al., 2005), and hydrogen bonds were constrained using the SHAKE algorithm (Ryckaert et al., 1977). Finally, the Nose–Hoover thermostat and MTK barostat (Martyna et al., 1994) were used to control the temperature and pressure during NPT dynamics on Anton2 using optimized parameters set by the *Multigrator* integrator (Lippert et al., 2013) of the machine.

### Trajectory analysis

We analyzed the trajectory primarily with VMD (William et al., 1996) and MDAnalysis (Michaud-Agrawal et al., 2011; Gowers et al., 2016). VMD was used to produce all the snapshots, and perform Hydrogen bond, RDFs, and packing defects analyses (Wildermuth et al., 2019). MDAnalysis was used to collect the raw data for the time series and histograms presented in this work, and in-house python scripts were used to further process the data and render all plots. Unless stated differently, all quantities are represented along with their standard error as computed from block averages during the listed time windows.

Cumulative plots were chosen to show lipid remodeling and recruitment by tracking the positions of the atoms in the lipid headgroup for a period of time. The size of this time window was determined to highlight differences between initial and final conditions on the membrane upon protein binding. The *xyz* coordinates were stored and accumulated for each lipid in one-nanosecond intervals for the determined time window. To show recruitment of inositol lipids, we rendered a scatter plot with a hue parameter of .5. To show height information, each z coordinate was selected and compared to the average z-coordinate of the first frame, $z_{o}$ , to find the relative position, $z_{f}=z-z_{o}$ . A scatter plot with z_f_ mapped to a color bar was plotted. Finally, to show the distribution of each lipid species per leaflet, we plotted a 2D density map in which the xy-plane was divided into a 2D grid and the number of points in each grid space was counted and plotted using a color bar.

Membrane lipids are free to move laterally, exposing regions of the hydrophobic core known as packing defects. These defects are enhanced when a protein binds and inserts into the membrane as it displaces lipids, making packing defects a good metric for protein insertion and membrane response. A method introduced by Wildermuth et al. measures the magnitude of the packing defects (Wildermuth et al., 2019). First, for a defined time window, images of the xy-plane are rendered with VMD. Hydrophobic atoms in the lipids are colored in yellow and the hydrophilic headgroups in blue. Supplementary Figure S3 shows a single-frame snapshot as an example; the yellow regions in the image correspond to packing defects. Multiple snapshots are taken from consecutive frames in the trajectory; an artificial intelligence algorithm for image analysis in the OpenCV python library identifies contours and measures their area. The code provided also allows for computation of the packing defect area underneath the protein, i.e. in the region delimited by the projection of the protein in the xy-plane (local packing defects). In this work, packing defects are used as a complementary measure for protein insertion in the binding leaflet.

## Results

### Protein binding conformation

**FIGURE 1:**
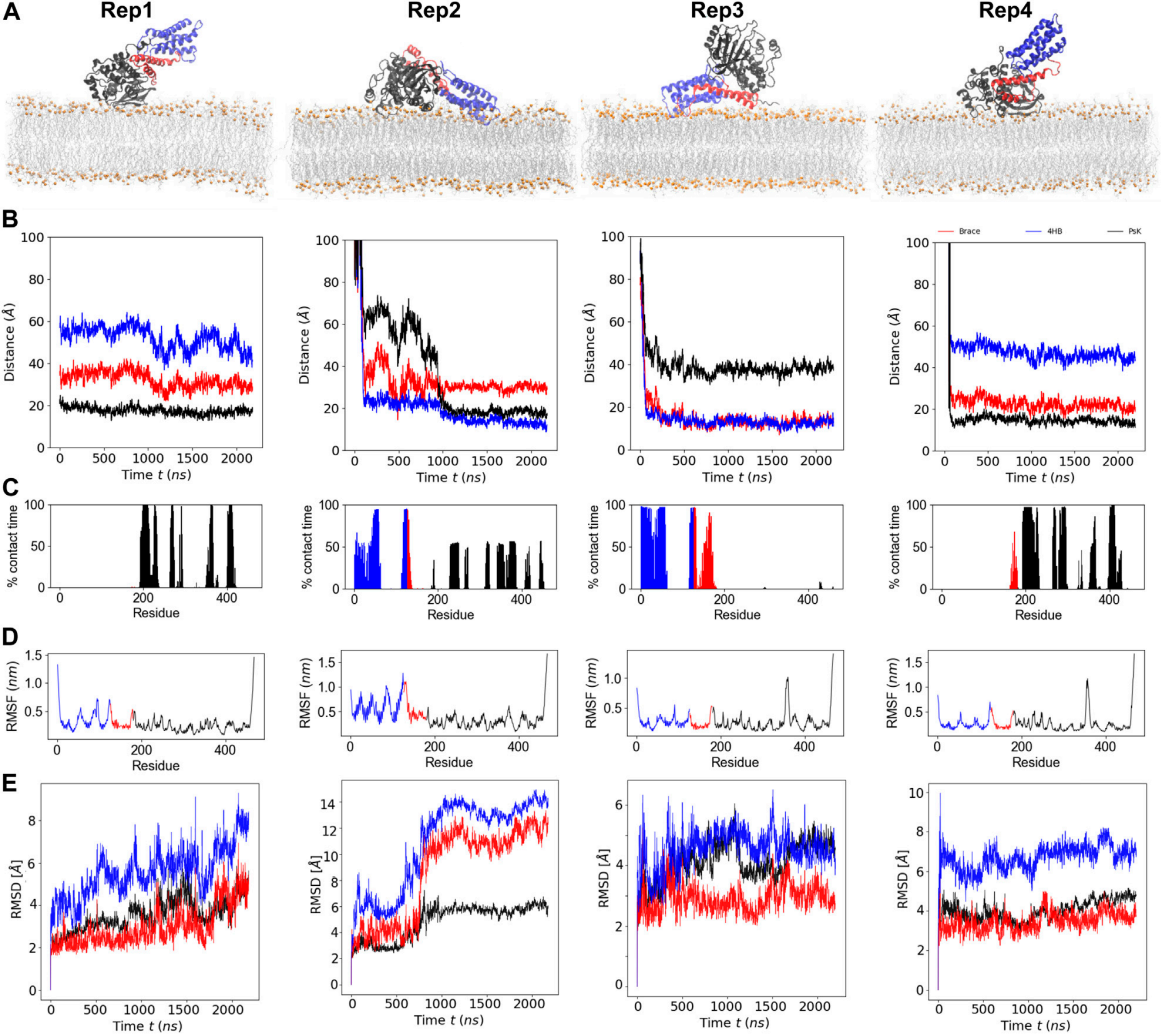
Binding conformation and dynamics of MLKL protein with a model membrane. **(A)** Final bound conformations of MLKL for each replica. The 4HB domain is shown in blue, the brace in red, and the PsK in black. Phosphate atoms of lipids are shown in orange, and fatty acid tails are shown in silver. **(B)** Center-of-mass distance of the $C_{\alpha }$ in each protein domain with respect to the phosphate groups in the binding leaflet. **(C)** Percent of time each residue in contact with any of the lipid species in the binding leaflet. **(D)** RMSF of protein residues. **(E)** Corresponding RMSD timeseries for each domain in the four replicas. Values corresponding to 4HB domain residues are indicated in blue, to the brace in red, and to the PsK in black in panels **(B–E)**, matching the domain colors in panel **(A)**.

MLKL binds the membrane within the first few hundred nanoseconds of simulation and remains bound the entire simulation. Figure 1A, shows the final bound conformation of the protein in each replica; despite the different initial orientations, common final bound states were found. Rep1 and Rep4 show a vertically bound conformation, with the PsK domain interacting with the membrane. Rep2 and Rep3 show the 4HB interacting with the membrane, which remains in contact with the bilayer until the end of the simulation. However, the PsK domain comes in contact with the bilayer after the first microsecond of simulation in Rep2, for a final horizontal bound conformation.

Figure 1B, shows the time series of the distance between the center of mass (COM) of the individual protein domains and the phosphate groups (P atoms) of the lipids in the binding leaflet; the COM of each domain was computed from its $C_{\alpha }$ atoms. As expected from the final conformations in Figure 1A, the PsK is the closest to the membrane in Rep1 and Rep4, followed by the brace, and no interaction of the 4HB with the bilayer. On the other hand, the 4HB is the first to contact the membrane in Rep2, the brace interacts with the membrane in the first half of the simulation, but then remains pointing towards the water when the Psk domain, shown in black, binds the membrane after the 1 μs mark. Finally, Rep3 shows the 4HB and the brace both interact with the bilayer at the same plane, while the PsK positions towards the water, at nearly 180^°^ with respect to its bound conformation on Rep1 and Rep4.

**FIGURE 2:**
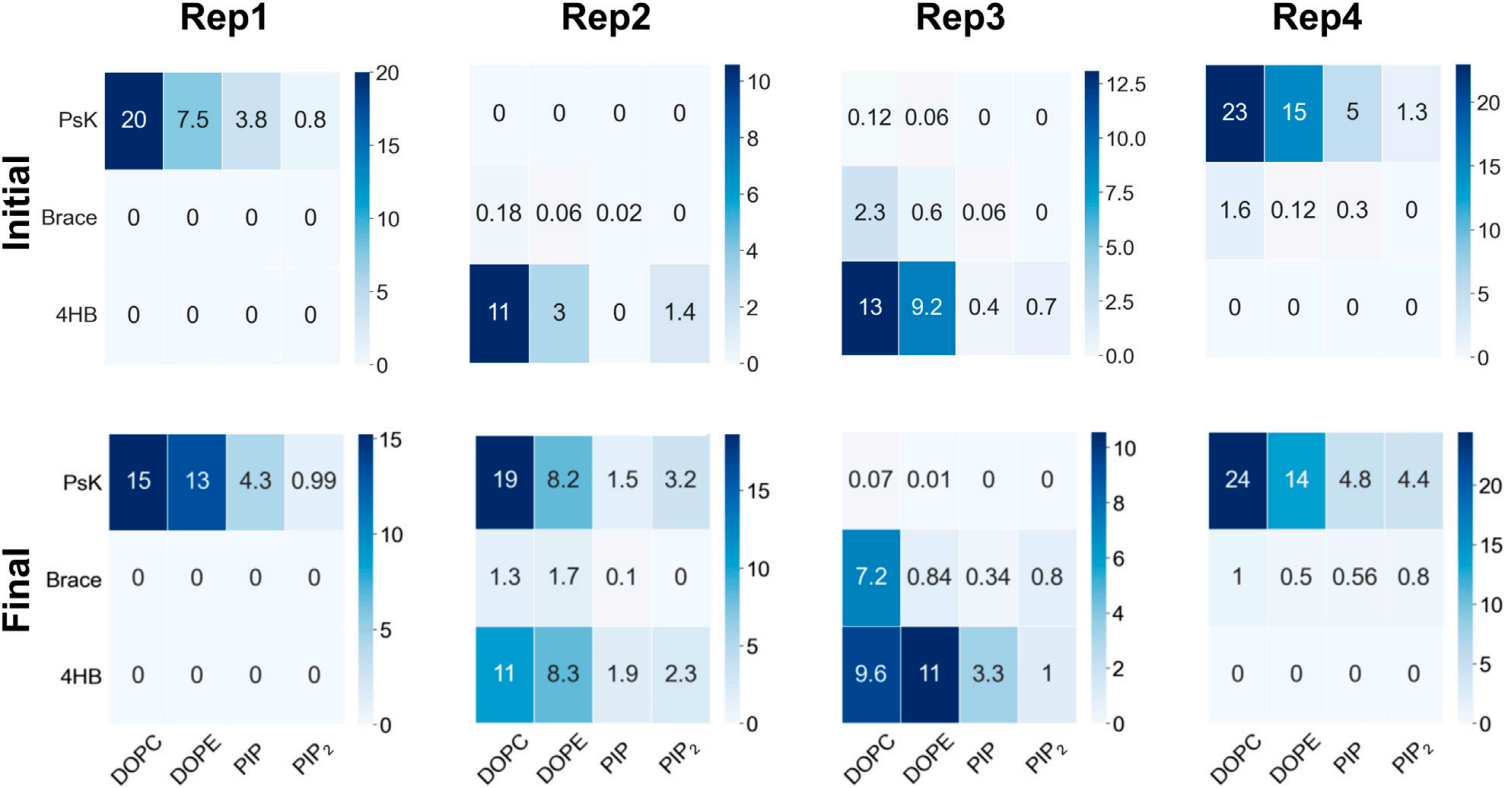
Number of contacts per protein domain versus lipid type during the initial 100ns upon protein binding (top row), and the last 200ns of trajectory, when lipid re-sorting has stabilized in response to protein binding (bottom row). The columns correspond to Rep1-Rep4 as labeled in the top of the figure. For all replicas, a protein-lipid contact is counted when atoms in a protein domain are within 12 Å of the phosphate atoms in the respective lipid species in the binding leaflet. Blue shade darkens as number of contacts increases.

A residue is considered in contact with the membrane when its $C_{\alpha }$ is located within 12 Å of lipid phosphate groups in the binding leaflet; unless mentioned otherwise, this cutoff is used for all contact analyses. The frequency of contact analysis per protein residue during the entire trajectory is presented as % contact time in Figure 1C. Rep1 and Rep4 show similar trends for the % contact time, with corresponding residues in the PsK domain in contact with the bilayer in both replicas. Note the brace domain does interact with the bilayer intermittently in Rep4, yet not permanently. Conversely, in Rep2 and Rep3, the frequency of contact is higher for residues in the 4HB domain. The main difference between these two replicas is that the PsK domain in Rep2 does interact with the membrane in the second half of the trajectory, adopting a fully horizontal position after 1 μ s of simulation. Some 4HB residues detach from the membrane as the PsK forms new contacts; Figure 2, Final stage, shows greater number of PsK-lipid contacts in Rep2 compared to PsK-lipid contacts in Rep3.

Figure 1D shows the root mean square fluctuations (RMSFs) of protein residues averaged over the total trajectory time. Rep3 and Rep4 exhibit a spike for residues surrounding residue 350 in the PsK domain (increased fluctuations in this region), while Rep2 exhibits larger fluctuations in the 4HB instead. Additionally, Figure 1E shows the root mean square displacement (RMSD) for each replica over the full trajectory. From this figure, it is evident there are no major conformational changes in the protein for Rep1, Rep3, and Rep4, which maintain their vertically bound conformation upon initial binding. Rep4 is the most stable of the four, as it barely changes over time with respect to is initial conformation. Rep1 experiences an increase in RMSD towards the end of the simulation, but the new conformation is stable for the last 200 ns of the trajectory. The PsK domain in Rep3 changes conformation after the first microsecond of the simulation, which was maintained for nearly 500 ns. There is a decrease in the RMSD of the black curve at this timepoint; however, the change is reverted and the RMSD returns to the value of the initial bound conformation for the rest of the trajectory.

The most interesting set of RMSD curves is that of Rep2 (Figure 1E), the only trajectory to exhibit both vertical and horizontal bound conformations. Upon initial vertical binding by the 4HB, the protein is stable with no shifts in conformation for nearly 500 ns. There is a noticeable increase in the RMSD of all three protein domains between 700 and 1,000 ns timepoints in the trajectory. Of the three domains, the brace is the one to show the sharpest change in configuration as it interacts with the bilayer (see the red curve in this plot). Following the same trend, the PsK domain also has a large conformational change as the protein lays horizontally on the membrane surface. The next section discusses changes in the lateral organization of lipids in response to the bound protein.

### Lipid contacts

As MLKL protein approaches the membrane, it interacts with specific lipid species in the binding leaflet, with a distinctive preference depending on the bound conformation. Figure 2 shows contact heatmaps for each lipid species in our model upon initial protein binding, and during the last 200ns of trajectory: DOPC, DOPE, PIP, and PIP_2_ with each protein domain (4HB, Brace, PsK). The overall number of contacts is higher with DOPC and DOPE in all cases, as expected, given their relative compositions in the membrane. PIP and PIP_2_ have fewer total number of contacts with the protein due to their relative abundance compared to other lipid species. However, as summarized in Figure 2, PIP and PIP_2_ co-localize to the protein binding site and increase their concentration rather notoriously. The following section expands on evidence of inositol recruitment to the protein binding site as evidenced by hydrogen bonding and 2D lipid density maps.

### Protein-lipid interactions: Hydrogen bonding

**FIGURE 3:**
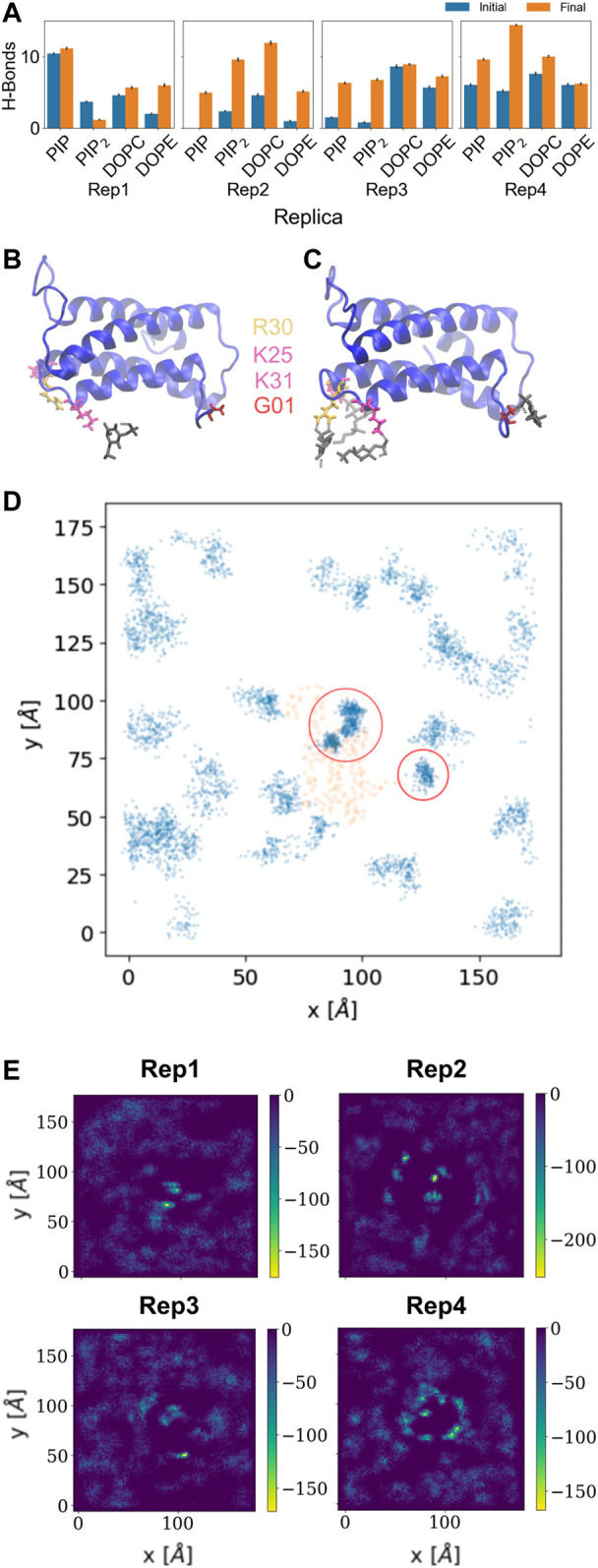
Hydrogen bonds and lipid recruitment upon MLKL binding. **(A)** Bar plots of the total number of hydrogen bonds between protein and lipids for each replica during the first 500 ns (in blue) versus the last 500 ns (in orange). Close-up of hydrogen bonds between PIP lipids and the 4HB domain at: **(B)** 200 ns, and **(C)**. 2000 ns. The 4HB domain is shown in blue, PIP lipid headgroup in black, R30 residue in yellow, K25 and K31 in pink, and G01 in red. **(D)** Cumulative plot of the lateral distribution of PIP lipids as estimated from the location of the lipid phosphate atoms for the last 200 ns. Blue points indicate the phosphate atom positions in the xy-plane, while orange points show representative positions of the atoms of the protein domains in contact (4HB and Brace). Panels **(B–D)** show analysis for Rep3, the replica of focus as discussed in subsequent sections. Similar plots for the remaining replicas are found in the SM, Supplementary Figure S6. **(E)** 2D surface charge distribution maps for the binding leaflets of each replica averaged over the last 500 ns of simulation. The cumulative negative charge is shown by the yellow/green regions, clearly localized in regions that correspond to the protein binding sites in each replica. The darker regions correspond to DOPC/DOPE-rich regions, with overall net charge of zero.

The hydrogen bond analysis shown in Figure 3A was performed with a donor-acceptor distance of 3.2 Å and a cutoff angle of 30° on the initial and final 500 ns of the trajectory using VMD. Figure 3A and Supplementary Table S1 summarize this analysis; the final number of hydrogen bonds between the protein and inositol lipids is highest for Rep1 and Rep4, compared to much lower net number of hydrogen bonds with DOPC or DOPE lipids. Figure 3A and Supplementary Figure S4 show the number of hydrogen bonds increases consistently across replicas for inositol lipids, Rep1 being the one where PIP has the highest number of hydrogen bonds with the protein. Similarly, PIP_2_ has larger number of hydrogen bonds as the simulation advances, except for Rep1; in all other cases, PIP_2_ is the species with highest increase in hydrogen bonds as the simulation progresses. Supplementary Figure S5A shows final snapshots of all four replicas with PI lipids, shown in red and blue, and cholesterol, shown in yellow, underneath the protein. Taken together, these results suggest lipid resorting patterns upon protein binding leave a specific lipid fingerprint at the protein binding site.

Figures 3B, C show examples of residues that form hydrogen bonds with PIP lipids, most of which are positively charged arginine and lysine residues. Specifically, G01, K25, R30, and K31 are highlighted. Figure 3D further shows the 2D cumulative density of PIP lipids on the membrane plane over the last 200 ns. The red circles show regions with greater lipid density that match the location of the protein, shown as orange scatter. Supplemenyary Figures S6D–F show similar density maps for PIP lipids for the remaining replicas, where we find similar patterns. Furthermore, Supplementary Figure S6G shows the time progression of PI lipids under and around the protein binding site; enriched PI lipid regions are linked to charged regions as shown for all replicas in Figure 3E. These plots show highly negative charged regions at and around the protein binding site, and correspond PIP and PIP_2_ enriched zones. For instance, Figure 3E for Rep4 shows a charged ring that matches the binding site.

### Membrane response: Lipid packing defects

**FIGURE 4:**
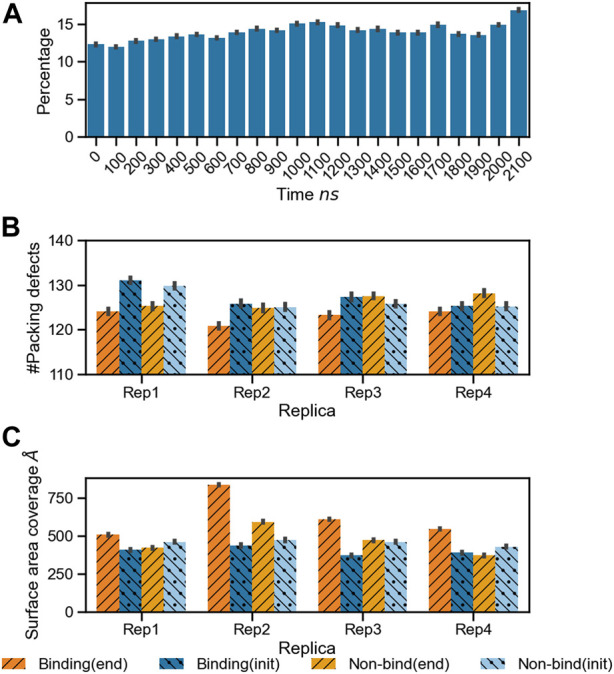
Changes in lipid packing defects in the immediate environment due to MLKL binding. **(A)** Bar plots for the percentage area of lipid packing defect at the protein binding site with respect to the projected protein surface area $\%=100\cdot \left(local.defects/protein.area\right)$ . Bars are plotted in time windows of 100 ns for the full trajectory of Rep3, and show a net increase in packing defects. (error bars indicate standard error). **(B)** Number of lipid packing defects per leaflet at the beginning (blue tones with left stripes and dots), and end (orange tones with right stripes) of the simulation. **(C)** Total surface area of packing defects per leaflet at the beginning (blue tones with left stripes and dots) and end (orange tones with right stripes and dots) of the simulation. Darker blue and orange represent the binding leaflet, and lighter hues show data for the non-binding leaflet.

As the protein interacts with the membrane, it influences the surface topology and lipid packing. We computed the lipid packing defects on the membrane prior to protein binding, and at the end of the simulation, when at least one microsecond of stable binding and subsequent lipid sorting around the protein has taken place. Figure 4A shows the percentage of surface area covered with lipid packing defects below the projected area of the protein during the trajectory for Rep3 (local packing defects, as described in the methods section). The area covered by the packing defects underneath the protein increases over time, correlating with protein insertion as verified by depth of bound residues in the binding leaflet (see Supplementary Figure S7). Figures 4B, C show the number of lipid packing defects and respective surface area coverage per leaflet at the beginning and end of the simulation with their associated standard error. Interestingly, while Rep1 and Rep4 do not exhibit significant changes in the number of packing defects between leaflets at the beginning vs. the end of the trajectory, the surface area coverage does change, with larger values in the binding leaflet. This is accentuated in Rep2 and Rep3, which show the most interesting behavior in terms of bound conformation and insertion of the 4HB past the lipid headgroup region (see Figures 1A, 7; Supplementary Figure S7). This fact is counterintuitive because the PsK domain, which binds the membrane in Rep1 and Rep4, is larger than the 4HB domain; yet, it does not insert as deep as 4HB (Supplementary Figure S7).

### Membrane response: Surface topology

**FIGURE 5:**
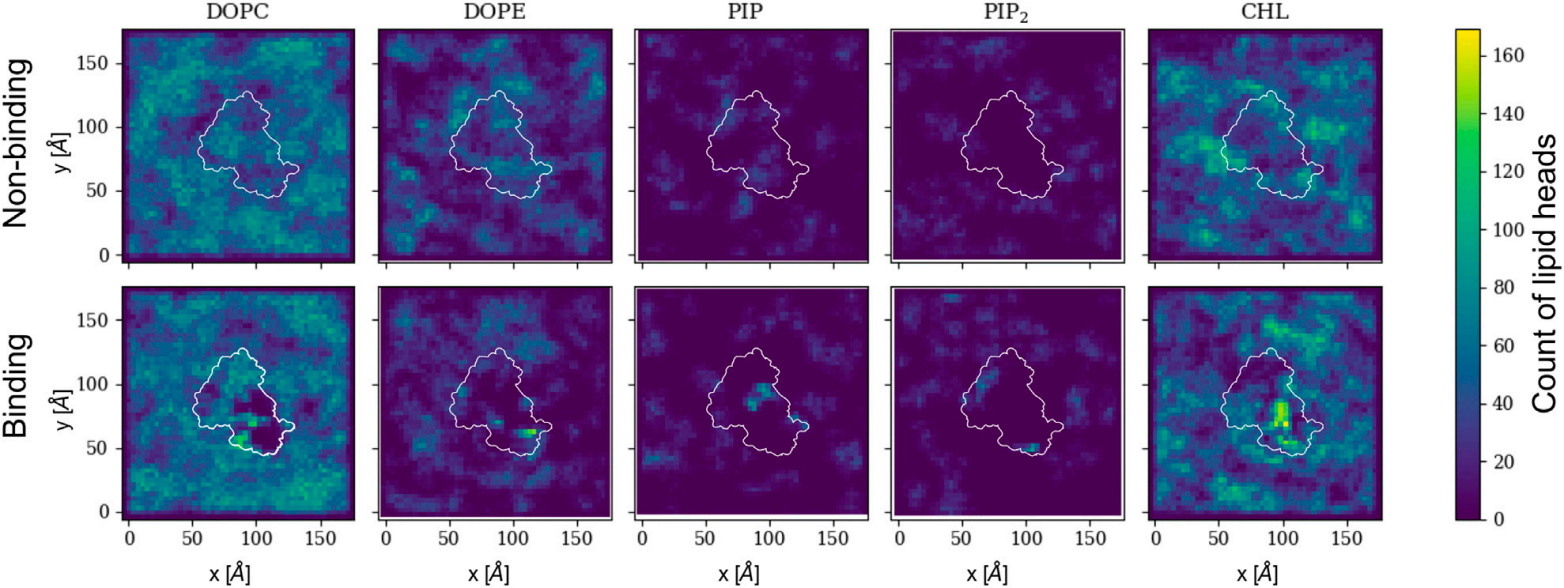
2D cumulative histograms for the last 500 ns of Rep3 trajectory. Top row corresponds to observations for non-binding leaflet, and the bottom row for the binding leaflet. White contours show a representative projection of the protein and the color bar is the cumulative number of lipids in each square of the 2D histogram, represented by either their P atom or O3 for cholesterol. Color intensity changes from dark blue to bright yellow as concentration of lipid head atoms increases.

The protein bound conformation directly influences the local lipid environment and, consequently, the surface topology. Figure 5 shows 2D histograms of the cumulative distribution of each lipid species per leaflet for the last 500 ns of the trajectory. Rep3 is shown as reference since it exhibits the deepest 4HB insertion across all replicas. This is in agreement with multiple studies indicating the role of 4HB is for retention and insertion of MLKL into the PM (Dondelinger et al., 2014; Su et al., 2014; Wang et al., 2014). Each cumulative histogram was generated by mapping the membrane onto a grid and counting the number of phosphate groups in each zone. The DOPC map, for example, shows a more uniform distribution of these lipids in the non-binding leaflet; whereas, there is clear depletion of DOPC directly underneath the protein binding site (see corresponding plot in the bottom row). On the other hand, PIP and PIP_2_, present at lower concentrations than DOPC, have a sharp increase around the protein site in the binding leaflet, shown in bright green/yellow in the map. This striking effect is also observed in cholesterol, which colocalizes underneath the protein binding site in the binding leaflet, and around the protein in the non-binding leaflet. Note that the cholesterol enrichment underneath the protein matches with the DOPC-depleted zone in the same leaflet. Similar 2D histograms for the other replicas are shown in Supplementary Figure S8.

**FIGURE 6:**
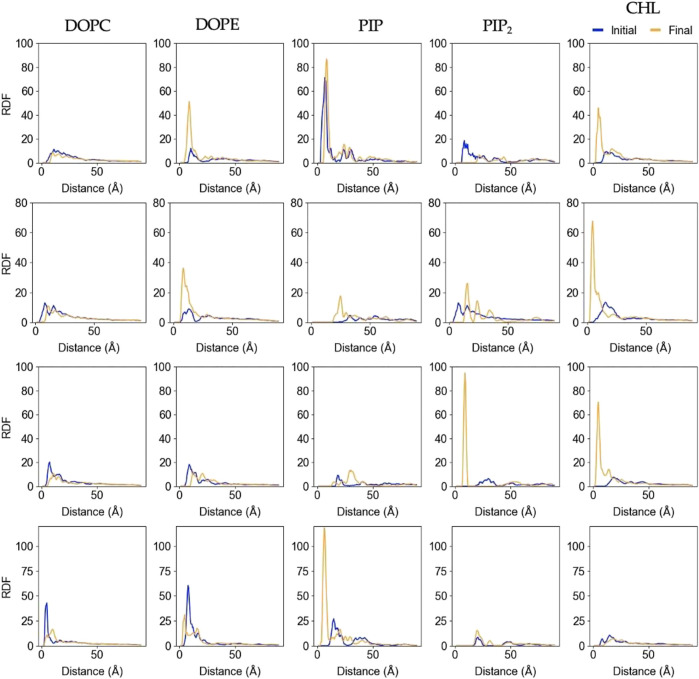
Lipid-Protein RDFs for each lipid species upon protein binding and at the end of the trajectory. The analysis was performed between the C_α_ atom of the deepest inserted protein residue in each replica (Rep1: K268, Rep2 and Rep3: Q53, and Rep4: G406), and the phosphorus (P) or hydroxyl oxygen (O3) of the lipids and cholesterol molecules, respectively. Initial curve is the averaged behavior during the first 50ns upon protein binding (shown in blue), and final is the average behavior over the last 100ns of simulation (shown in orange). Each row corresponds to Rep1-4, and each column corresponds to the lipid species listed at the top.

Figure 6 shows changes in the lateral lipid organization through protein-lipid Radial Distribution Functions (RDF). These were calculated by using the $C_{\alpha }$ atom of the deepest inserted protein residue in each replica as a reference point (K268 for Rep1, Q53 for Rep2 and 3, and G406 for Rep4 – see Supplementary Figure S7 for details on identifying these residues), and the phosphate or hydroxyl oxygen atoms of phospholipids and cholesterol, respectively. Each plot compares the RDF for the first 50 ns upon protein binding and the last 100 ns of the trajectory. Rep1 shows a slight increase in cholesterol and a noticeable decrease in PIP_2_ near the bound protein at the end of the trajectory.

In most cases, the likelihood of observing cholesterol and PIP lipids under the protein or closer to the deepest inserted residue is higher at the end of the simulation compared to the beginning, which is also depicted in Supplementary Figure S5A. The RDFs for DOPC and DOPE retain the location of the first solvation shell; DOPC experiences little to no change, but DOPE has higher relative abundance at the end in most of the cases. Rep2 exhibits the most interesting change for PIP_2_ lipids, as the final RDF shows three distinctive shells. Note that Rep2 is the only replica that shows the protein interacting with the bilayer in a fully horizontal fashion, in which all the domains interact to some extent with the bilayer. The bottom row of Figure 2 and Supplementary Figure S5A show PIP interaction with the protein. Supplementary Figure S9 shows the corresponding RDF analysis for lipid-lipid interactions on the membrane plane of the binding leaflet. Results for all replicas show little to no change in the lateral distribution of DOPC, DOPE, and cholesterol species on the entire binding leaflet. However, the height and width of the solvation shells for PIP and PIP2 species do change; in this case, Rep4 shows two distinct solvation shells for PIP and PIP_2_ lipids at the end versus the beginning. Additionally, Rep2 shows an inward shift and higher probability for the first solvation shell of PI lipids at the simulation end versus the beginning.

**FIGURE 7:**
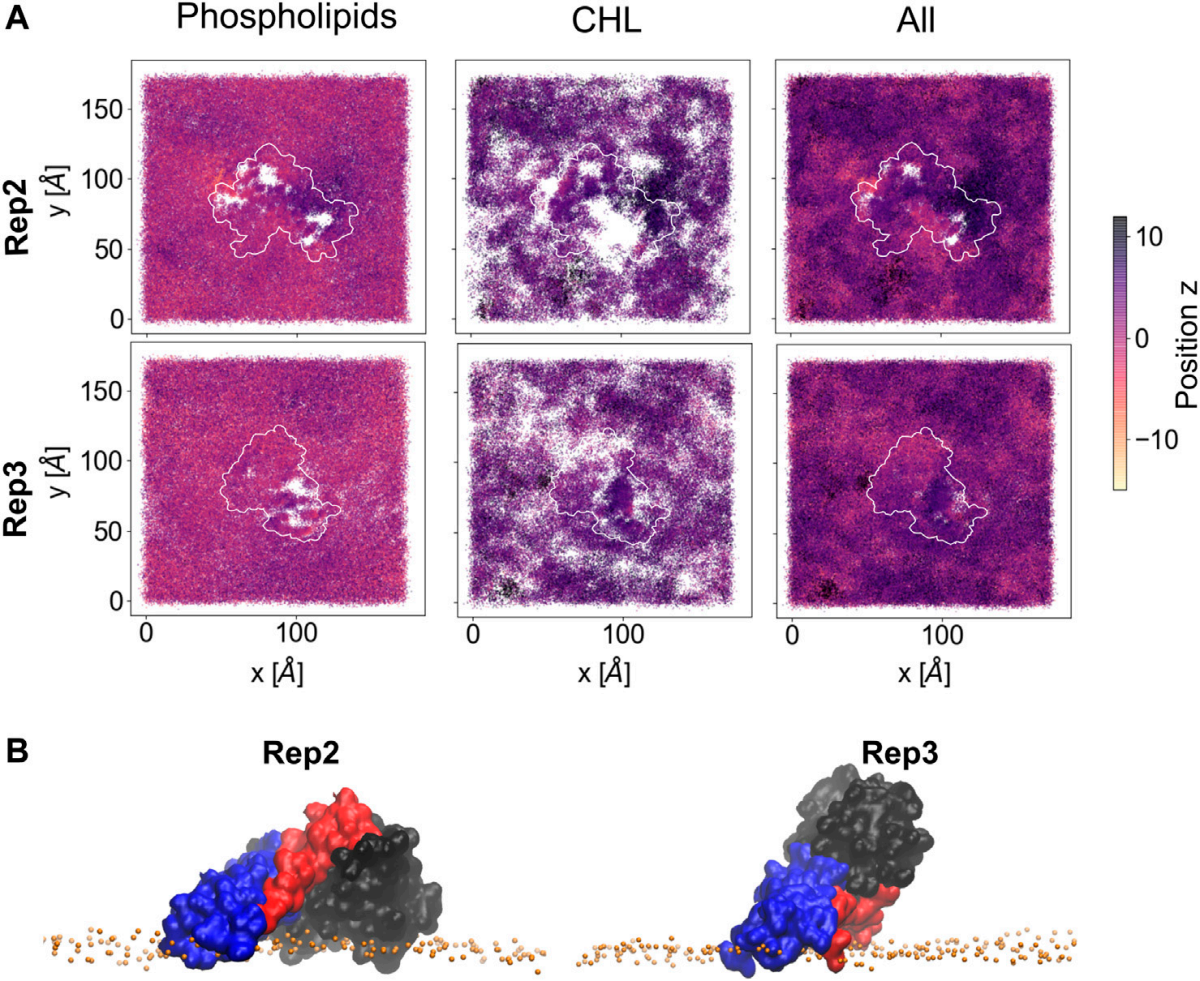
Membrane deformation due to protein binding. **(A)** Cumulative membrane height (z-coordinate) during the last 500 ns of simulation for Rep2 (top row) and Rep3 (bottom row). The color maps show the relative position of the lipid phosphate atoms (left), cholesterol hydroxyl group atoms (middle), and both types of atoms (right) on the binding leaflet. The relative positions are computed with respect to their initial coordinates in the analysis period (i.e., the first frame of the last 500ns of trajectory). Color intensity changes from pale yellow to dark purple as z position of atoms increase; white patches indicate absence of headgroup atoms. **(B)** Close up of the bound protein for Rep2 and Rep3, showing different domains inserted past the phosphate atoms in the membrane, shown as orange spheres. The 4HB is shown in blue, brace in red and PsK in black. Similar plots for Rep1 and Rep4 in Supplementary Figure S10.

Lipid sorting directly impacts the topology of the membrane surface; Figure 7 shows the cumulative changes on the topology of the membrane surface during the last 500 ns of the trajectory for Rep2 and Rep3, respectively, as these replicas exhibit protein insertion past the lipid headgroup region. These changes are calculated using the first frame of the selected trajectory as the reference point. Supplementary Figure S10 shows the corresponding plots for Rep1 and Rep4; the latter shows a small indentation of the PsK domain in the bilayer, but not as pronounced as the displacement of lipids around the 4HB in Rep3. In Rep2 and Rep3, the 4HB domain inserts past the lipid headgroup region, and cholesterol can mitigate displacement of phospholipids by filling the space those lipids occupied without pushing the protein away.

## Discussion and conclusion

Four replicas were run starting from different MLKL protein positions near a membrane model to characterize the protein binding mechanism and associated membrane response. Our results show MLKL is attracted to the membrane *via* electrostatic interactions. Then, as the protein binds the membrane, it remodels the local lipid environment by depleting DOPC, DOPE, and recruiting PIP, PIP_2_, and cholesterol consistent with previously established experimental models (Dondelinger et al., 2014). Local remodeling of lipid composition depends, to a large extent, on the protein domain that binds the membrane. When the 4HB binds the membrane, it can insert past the phosphate region of the lipids, increasing the number of packing defects as it displaces the lipid headgroups and interacts with the hydrophobic core. Bound conformations with the 4HB interacting with the bilayer align to what has been proposed in the literature for MLKL during PM permeabilization.

Dondelinger et al. (2014) propose the 4HB as the executor of necroptosis, where the process is driven by interactions between highly conserved positive residues in the first two alpha helices in the 4HB and PIP lipids. More recently, experimental and simulation works suggest that the brace domain is an active player in the process of association of MLKL to lipid membranes (Yang et al., 2021; Sethi et al., 2022b). For example, it is reported that interactions between positively charged residues in MLKL and the membrane pull the brace away from 4HB for activation of this domain in human MLKL (Sethi et al., 2022b). Quarato *et al* propose a mechanism of initial recruitment of MLKL to the plasma membrane *via* low affinity interactions between positive residues on the helices of 4HB and membrane lipids, bringing the brace in closer proximity to the membrane – similar to what was observed in Rep2 and Rep3 in this work. This unmasks further positive residues on the 4HB, leading to enhanced interaction with PIPs, also reproduced in our trajectories as shown in Figure 3, Supplementary Figures S4, S6. In our simulations, R30 exhibits hydrogen bonding with PIPs, this residue has also been identified as critical for binding to the membrane and stabilizing the interaction of the brace domain (Quarato et al., 2016). In line with these observations, Rep3 seems the most likely scenario to represent the interaction of MLKL with the plasma membrane in the cellular environment *via* both the 4HB and the brace.

The PsK domain is well known to interact with other proteins such as RIPK3 during necroptosis and act as a conformational-change switch that activates MLKL after undergoing phosphorylation (Petrie et al., 2017). Apart from this, not much is known about its interactions with membrane lipids, or if PsK-lipid interactions are relevant in the context of necroptosis and membrane disruption. Our results show Rep1 and Rep4 interact with the membrane through the PsK domain stably during the entire trajectory (see Figure 1E), and with Rep2 after 1 μ s. Our analyses examining the local lipid distribution, hydrogen bonding, and membrane response do not give direct strong evidence of a preferred binding domain. However, the number of contacts between each domain and specific lipid species does lead to a distinctive lipid fingerprint and local lipid distribution (see Figures 2, 3, 5; Supplementary Figures S9, S10). Based on the simulations presented here, it seems possible that a cooperative effect for MLKL binding and oligomerization could lead to membrane permeabilization; however, this event is not seen within the scope of our simulations in this study. Given that all 3 domains can interact with the membrane, it is possible that each domain contributes to specific lipid interactions that aid the process of membrane remodeling, and eventual bilayer disruption and permeabilization in a cooperative manner.

We observe the protein binds the membrane in all replicas well within the first 200 ns of simulation. Rep2 further exhibits a change in bound conformation after a microsecond of simulation and stable binding in a vertical conformation. The protein is able to turn and remain horizontally at the membrane interface with both the PsK and 4HB interacting with the lipids. In all replicas, the lipid distribution at the protein binding site changed depending on the protein domain bound at the membrane. For example, Rep3 experiences a drastic change in local lipid composition when the brace domain contacts the membrane surface. The DOPE:PIP_2_ ratio changes from 5:1 to ∼1:1; additionally, at the 4HB-membrane contact site the DOPC:PIP_2_ ratio changes from 10:1 to ∼10:3. The initial ratios are based on the initial lipid composition, whereas the final ratios are extracted by counting the lipid species underneath the protein and determining their relative composition at the protein binding site. The change in lipid ratio is a clear indicator of local lipid redistribution directly modulated by the protein residues that bind the membrane. This distinct lipid fingerprint in the case of MLKL seems to result mainly from electrostatic interactions of positively charged residues and negatively charged lipid headgroups. The bottom panels in Figure 3 further show a distinct distribution of charge around the protein, in the ring-like 2D density maps right at the edge of the protein binding site.

Some of the key charged residues that interact with the membrane are shown in Figure 3. Notably, the positively charged residue K31, located in the second helix of the 4HB domain in the mouse model (4BTF) studied in this work, is conserved in human MLKL. Experiments with human MLKL have shown that the positively charged residues 22-35 are facilitators of MLKL oligomerization and recruitment to membranes as they interact with PIP lipids (Dondelinger et al., 2014). Our simulations with the murine protein model agree with a conserved behavior of these residues across both human and mice MLKL. There are differences in the report of relevant residues between the two orthologs; one work suggests that the mouse model associates with the membrane *via* residues found in the third and fourth helices of the 4HB, in contrast to the human model (Sethi et al., 2022b). While initial interactions of MLKL in our simulations are due to electrostatics, there is noticeable recruitment of PIP and PIP_2_ lipids to the protein binding site, further stabilized by hydrogen bonding and displacement of net neutral lipids like DOPC to the membrane bulk. This is shown in Figure 5, the cumulative lipid density plots for different lipid species that show the regions where these are enriched or depleted on the membrane plane (Petrie et al., 2018; Murphy, 2020). The binding conformation of even a single protein is able to alter local lipid distribution, generating a distinctive lipid fingerprint and lateral organization patterns (see Figure 7, RDFs). Cumulative plots of the lateral distribution of PIP lipids in Figure 4D and Supplementary Figures S3D–F further support this premise, showing stronger concentration of PIP lipids near the protein over time in agreement with experiment (Dondelinger et al., 2014).

The formation of a characteristic lipid fingerprint upon MLKL binding also impacts membrane lateral packing and surface topology, shown in Figures 4, 7A, respectively. The relationship between protein binding and distribution of packing defects in the binding and non-binding leaflets is not trivial; we observe distinctive behavior for the different protein bound domains across our replicas. Packing defects underneath the protein were found to be significantly larger in Rep2 and Rep3 (Figures 4B, C), where the 4HB interacts with the membrane. This domain inserts past the phosphate region of the binding leaflet, resulting in a rearrangement of lipid packing. The overall number of lipid-packing defects decreases, but their overall surface coverage increases (Figures 4B, C); suggesting smaller packing defects merge into larger ones as lipid sorting and recruitment to the protein binding site progress. In contrast, the increase of packing defects surface area right below the protein in Rep1 and Rep4 is rather subtle, and corresponds to a small or no insertion into the membrane. These results agree with previous observations in the literature that identified the 4HB as the killer domain (Dondelinger et al., 2014; Hildebrand et al., 2014).

From Figures 5–7, it is evident that cholesterol is attracted to the protein in the binding leaflet, creating a distinctive fingerprint that differs from the non-binding leaflet. Accumulation of cholesterol is known to increase order in the membrane hydrophobic core and decrease membrane fluidity (Czub and Baginski, 2006). In the context of membrane permeabilization, accumulation of cholesterol under MLKL binding sites in the inner leaflet of the PM could potentially lead to a more fluid outer leaflet in the PM that allows easier permeation of small molecules around the protein or oligomers. Alternatively, the clustering of cholesterol near MLKL may be related to a necroptosis-independent role of the protein in lipid trafficking. Though there is little in literature that discusses the effects of cholesterol accumulation in the PM during necroptosis, it is relevant for intracellular membranes. Death of atherosclerotic lipid plaques is caused by cholesterol accumulation in the endoplasmic membrane, which triggers the unfolded protein response and in turn, apoptotic pathways (Tabas, 2004). Additional studies would help determine if lipid sorting due to MLKL binding follows a cooperative effect, in which more protein units are attracted to the initial protein binding site due to the local lipid composition remodeling. From our current studies, limited to a single MLKL near the membrane and time scales that did not show disruption of the membrane, it seems plausible that oligomerization could be enhanced by lipid re-sorting caused by previous MLKL binding events (Flores-Romero et al., 2020).

Biochemical and lipidomic-based studies identified that phosphorylation of MLKL prior to plasma membrane association (Wang et al., 2014) or S-acylation of the protein can exacerbate membrane permeabilization (Parisi et al., 2017; Parisi et al., 2019; Pradhan et al., 2021); yet, how these modifications impact membrane permeability is not fully understood. The need of MLKL oligomers has been widely accepted in its mechanism to permeabilize the membrane during necroptosis; however, there are conflicting reports in the number of units that form the oligomer (Hildebrand et al., 2014). This work offers a basis for the study of membrane response and the specific lipid fingerprint that results upon binding of a peripheral membrane protein, specifically during initiation of MLKL driven mechanisms of cell death. The present work does not attempt to fully explain the process of protein-mediated permeabilization of the PM. Instead, it is geared towards characterizing the molecular mechanisms that may contribute to membrane remodeling and eventual disruption as a result of specific protein-lipid interactions. There is still much to explore in the context of MLKL-lipid interaction dynamics and how these shape the membrane surface topology, especially when multiple protein units are involved.

## Data Availability

Publicly available datasets were analyzed in this study. This data can be found here: https://www.rcsb.org/- 4BTF and https://www.uniprot.org/- Q8NB16. The four simulation trajectories will be available upon publication on the Anton2 site, https://antonweb.psc.edu/trajectories/Monje-Galvan/. In house analysis scripts using Gromacs, VMD, and MDAnalysis are available upon request.
