# Supplementary material for: Modeling the molecular fingerprint of protein-lipid interactions of MLKL on complex bilayers
Source: Front Chem. 2023 Jan 12;10:1088058. doi: 10.3389/fchem.2022.1088058 (PMC9877227; doi:10.3389/fchem.2022.1088058)
Supplement: Supplementary file 1 [file DataSheet1.pdf]

Supplementary Material

Modeling the molecular fingerprint of protein-lipid interactions of MLKL on complex bilayers.

Ricardo X. Ramirez,[1] Oluwatoyin Campbell,[1] Apoorva J. Pradhan,[2] G. Ekin Atilla-Gokcumen,[2] Viviana Monje-Galvan[1*]

[1]Department of Chemical and Biological Engineering, School of Engineering and Applied Sciences, University at Buffalo, Buffalo, NY, USA

[2]Department of Chemistry, College of Arts and Sciences, University at Buffalo, Buffalo, NY, USA

Table S1: Block average of hydrogen bonds for the last 1000 ns of trajectory showing values of PIP and PIP2 comparable or bigger than those for DOPC and DOPE.

Lipid	Rep1	Rep2	Rep3	Rep4
**PIP**	13.08±0.11	5.23±0.15	5.62±0.13	9.66±0.09
**PIP2**	1.46±0.04	10.51±0.28	6.56±0.10	13.26±0.14
**DOPC**	5.29±0.13	6.10±0.16	6.64±0.13	6.62±0.13
**DOPE**	5.47±0.12	12.73±0.26	8.66±0.13	9.75±0.13

MSA

The multiple sequence alignment result as produced by T-coffee.

```
T-COFFEE, Version_11.00 (Version_11.00)
Cedric Notredame
SCORE=978


sp|Q8NB16|MLKL_    :  96
4BTF_1|Chain       :  97
cons               :  97

sp|Q8NB16|MLKL_    -----MENLKHIITLGQVIHKRCEEMKYCKKQCRRLGHRVLGLIKPLEMLQDQGKRSVPSEKL
4BTF_1|Chain       GAMGSMDKLGQIIKLGQLIYEQCEKMKYCRKQCQRLGNRVHGLLQPLQRLQAQGKKNLPD-DI

cons                    *::* :**.***:*:::**:****:***:***:** **::**: ** ***:.:*. .:


sp|Q8NB16|MLKL_    TTAMNRFKAALEEANGEIEKFSNRSNICRFLTASQDKILFKDVNRKLSDVWKELSLLLQVEQR
4BTF_1|Chain       TAALGRFDEVLKEANQQIEKFSKKSHIWKFVSVGNDKILFHEVNEKLRDVWEELLLLLQVYHW

cons               *:*:.**. .*:*** :*****::*:* :*::..:*****::**.** ***:** ***** :


sp|Q8NB16|MLKL_    MPVSPISQGASWAQEDQQDADEDRRAFQMLRRDNEKIEASLRRLEINMKEIKETLRQYLPPKC
4BTF_1|Chain       NTVSDVSQPASWQQEDRQDAEEDG---------NENMKVILMQLQISVEEINKTLKQ-CSLKP

cons                .** :** *** ***:***:**          **:::. * :*:*.::**::**:*  . *


sp|Q8NB16|MLKL_    MQEIPQE-QIKEIKKEQLSGSPWILLRENEVSTLYKGEYHRAPVAIKVFKKLQAGSIAIVRQT
4BTF_1|Chain       TQEIPQDLQIKEIPKEHL-GPPWTKLKTSKMSTIYRGEYHRSPVTIKVFNNPQAESVGIVRFT

cons                *****: ***** **:* *.**  *: .::**:*:*****:**:****:: ** *:.*** *


sp|Q8NB16|MLKL_    FNKEIKTMKKFESPNILRIFGICIDETVTPPQFSIVMEYCELGTLRELLDREKDLTLGKRMVL
4BTF_1|Chain       FNDEIKTMKKFDSPNILRIFGICIDQTVKPPEFSIVMEYCELGTLRELLDREKDLTMSVRSLL

cons               **.********:************:**.**:**********************:. * :*


sp|Q8NB16|MLKL_    VLGAARGLYRLHHSEAPELHGKIRSSNFLVTQGYQVKLAGFELRKTQTSMSLGTTREKTDRVK
4BTF_1|Chain       VLRAARGLYRLHHSET--LHRNISSSSFLVAGGYQVKLAGFELSKTQNSISRTAKSTKAERSS

cons               ** ************:  ** :* **.***: *********** ***.*:*  :.  *::* .


sp|Q8NB16|MLKL_    STAYLSPQELEDVFYQYDVKSEIYSFGIVLWEIATGDIPFQGCNSEKIRKLVAVKRQQEPLGE
4BTF_1|Chain       STIYVSPERLKNPFCLYDIKAEIYSFGIVLWEIATGKIPFEGCDSKKIRELVAEDKKQEPVGQ

cons               ** *:**:.*:: *  **:*:***************.***:**:*:***:*** .::***:*:


sp|Q8NB16|MLKL_    DCPSELREIIDECRAHDPSVRPSVDEILKKLSTFSK------
4BTF_1|Chain       DCPELLREIINECRAHEPSQRPSVDGILERLSAVEESTDKKV

cons               ***. *****:*****:** ***** **::**:..:
```

Figure S1. Sequence alignment of the 4HB and brace residues in the mouse (PDBID: 4BTF) and human (Uniprot: Q8NB16) models. Conserved residues are listed with a start (*) in the *cons* line.

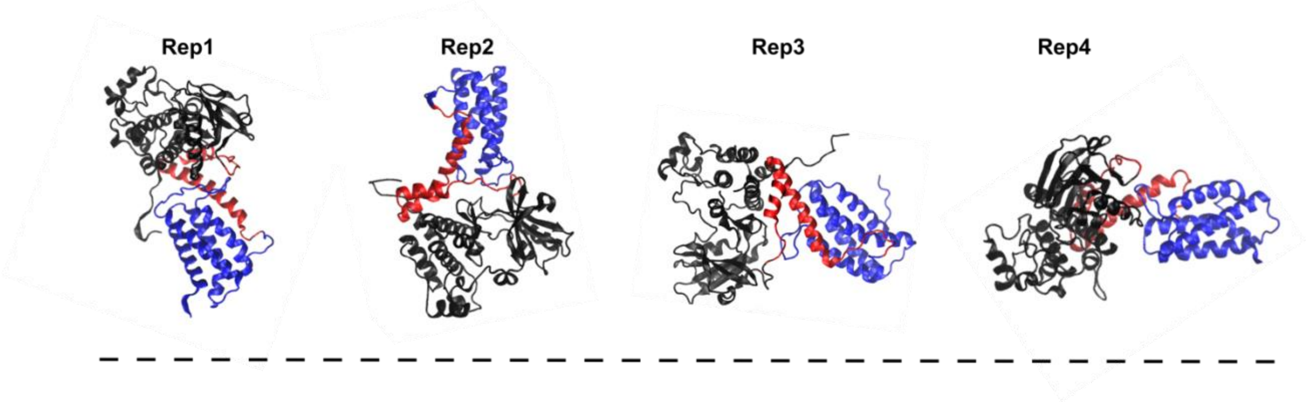

Figure S2. Initial orientations of MLKL with respect to the membrane surface, shown for reference as a dashed line. The four helical bundle (4HB) domain is shown in blue, the brace in red, and the pseudo-kinase (PsK) in black. These orientations were selected to prevent bias in the binding conformation. Given the nature of MD simulations, it is expected that the average protein-lipid interaction and binding behavior can be reproduced from multiple replicas that start from different configurations. That is, if the simulation is run for long enough time, and provided energy barriers are not significant for certain changes in protein conformations.

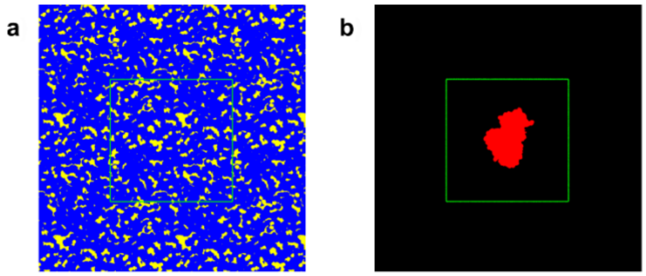

Figure S3. Sample snapshots of the packing defects analysis. **a.** Image generated on VMD showing the top view of the membrane (xy-plane) with the hydrophobic core in yellow and the hydrophilic headgroups in blue. **b**. Image generated on VMD with the top view of the protein red, this region is the protein projection on the membrane plane The green boxes show the boundaries of the main simulation cell, the image is generated taking into account periodical boundary conditions (regions outside the green box).

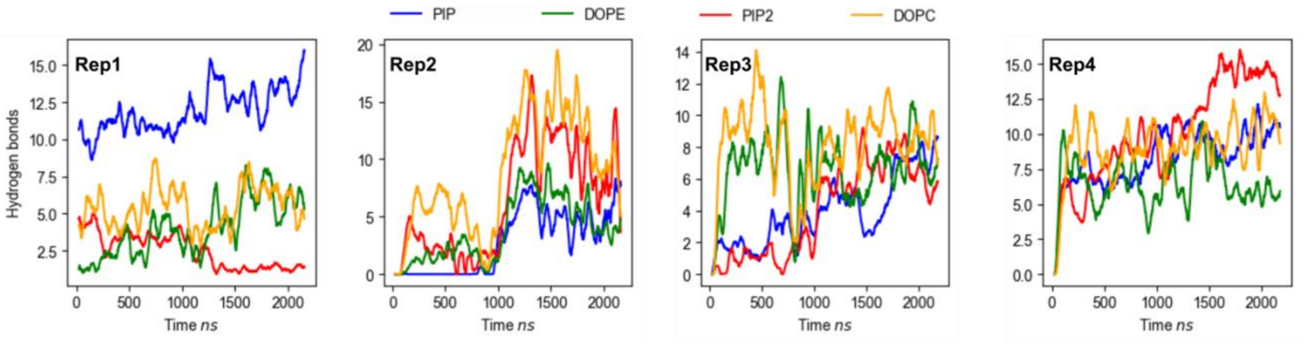

Figure S4. Hydrogen bonds timeseries for all replicas. Trends for PIP lipids are shown in blue, DOPE in green, PIP_2 in red, and DOPC in yellow. Note that PIP and PIP_2 are the curves that increase the most over time, suggesting PI lipids are recruited to the protein binding site, i.e., more interactions appear over time.

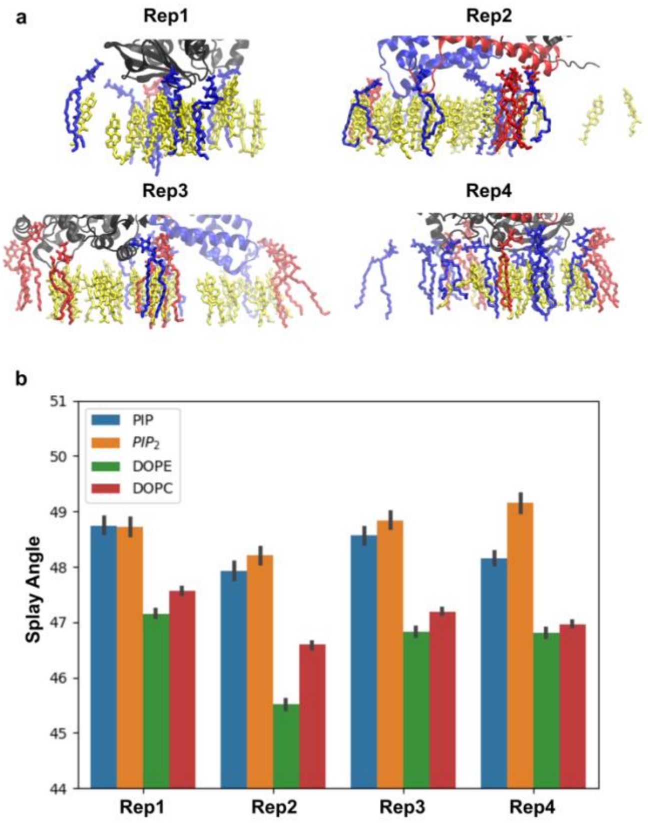

Figure S5. a. Final lipid conformation underneath the protein. Depending on the binding conformation, inositol lipids arranged in an orderly fashion below the protein binding site, both tails very closed to each other and nearly parallel to the membrane normal (z-axis in our simulations). **b.** Splay angle between lipid tails; PIP lipids are represented with blue, PIP_2 in red, and CHL in yellow. In Rep3 and Rep4, the inositol lipids arranged in a disorderly manner with a larger splay angle. DOPC and DOPE are omitted for clarity.

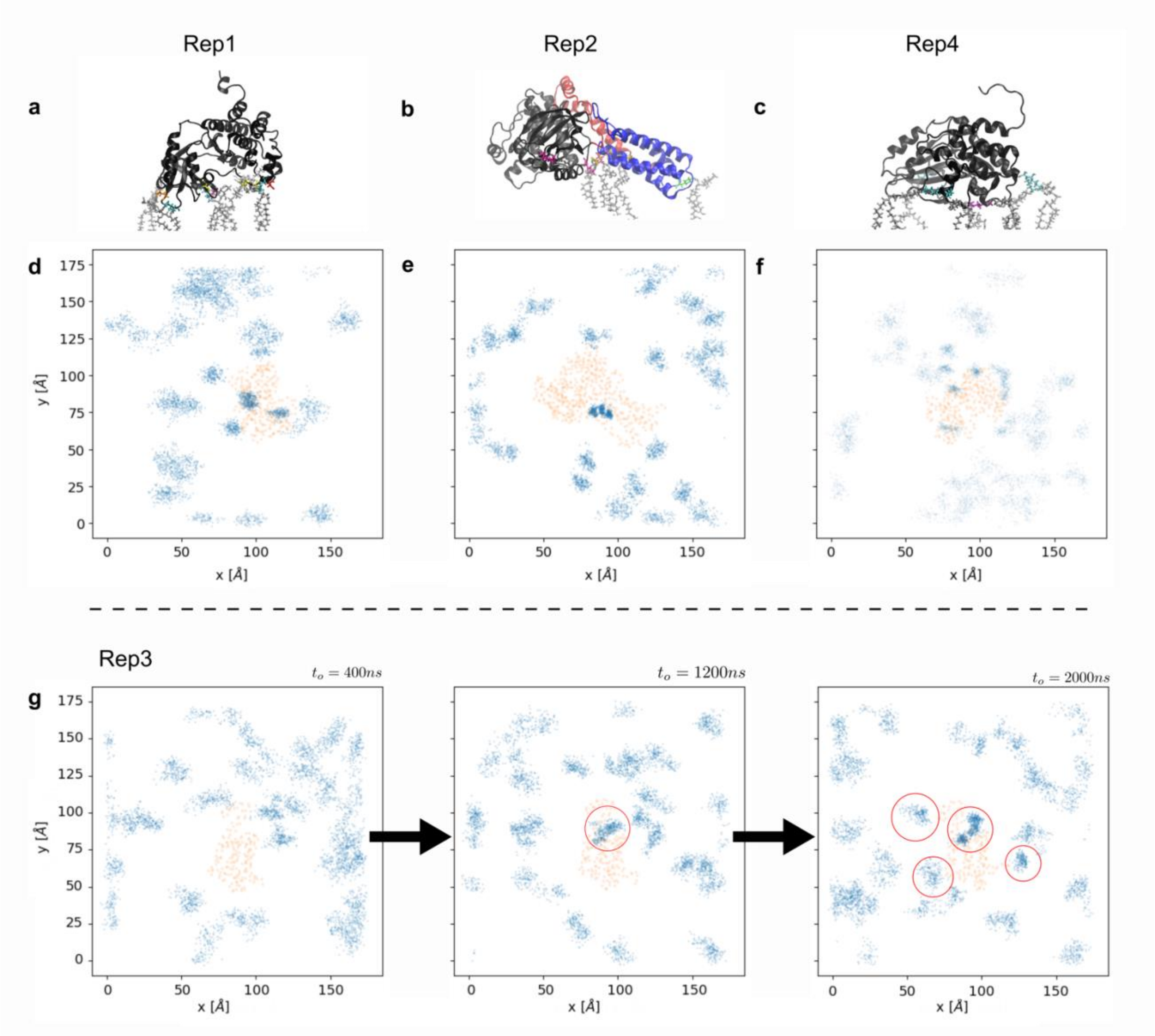

Figure S6. Lipid recruitment at the protein binding site, further stabilized by hydrogen bonding. Close-up of PIP lipids bound to the respective domain; **a.** Psk in Rep1, **b.** 4HB and PsK in Rep2, and **c.** PsK domain in Rep4. **d-f**. Cumulative plots computed for inositol lipids in the binding leaflet for Rep1, Rep2, and Rep4, respectively. Individual lipid positions were averaged over the last 200ns of simulation, as accounted based on the xyz-coordinates of the phosphorous (P) atom of the lipid molecules. The blue points indicate the position of the P-atoms in the binding leaflet, and the orange points show the positions of protein C_α. **g**. Cumulative plots of inositol lipids in Rep3 over 200ns starting at t_o (the top right corner of each plot). The time progression shows the recruitment of inositol lipids underneath the protein; darker blue indicates more density of inositol lipids underneath the protein (red circles highlight most notorious regions)

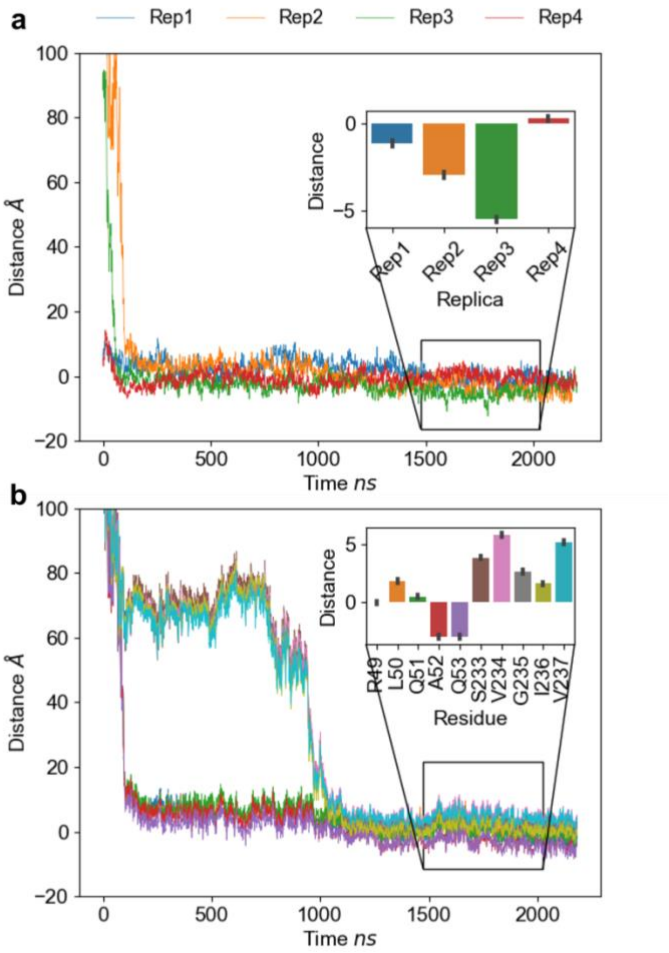

Figure S7: **a**. Distance of the residue with deepest insertion in each replica (Rep1: V268, Rep2 and Rep3: Q53, Rep4: G406). The inset plot shows a bar plot corresponding to the average distance of insertion for the last 500ns of the trajectory, showing that Rep2 and Rep3 are the replicas where we find the deepest insertion. **b**. Sample computation to determine the deepest inserted residue for Rep3; the analysis was done for all the residues that are inserted as identified with MDAnalysis, the inset plot shows the bar plot used to identify the deepest inserted residue from the equilibrated portion of the trajectory (1000-2000ns in this replica), Q53 and A52 in Rep3

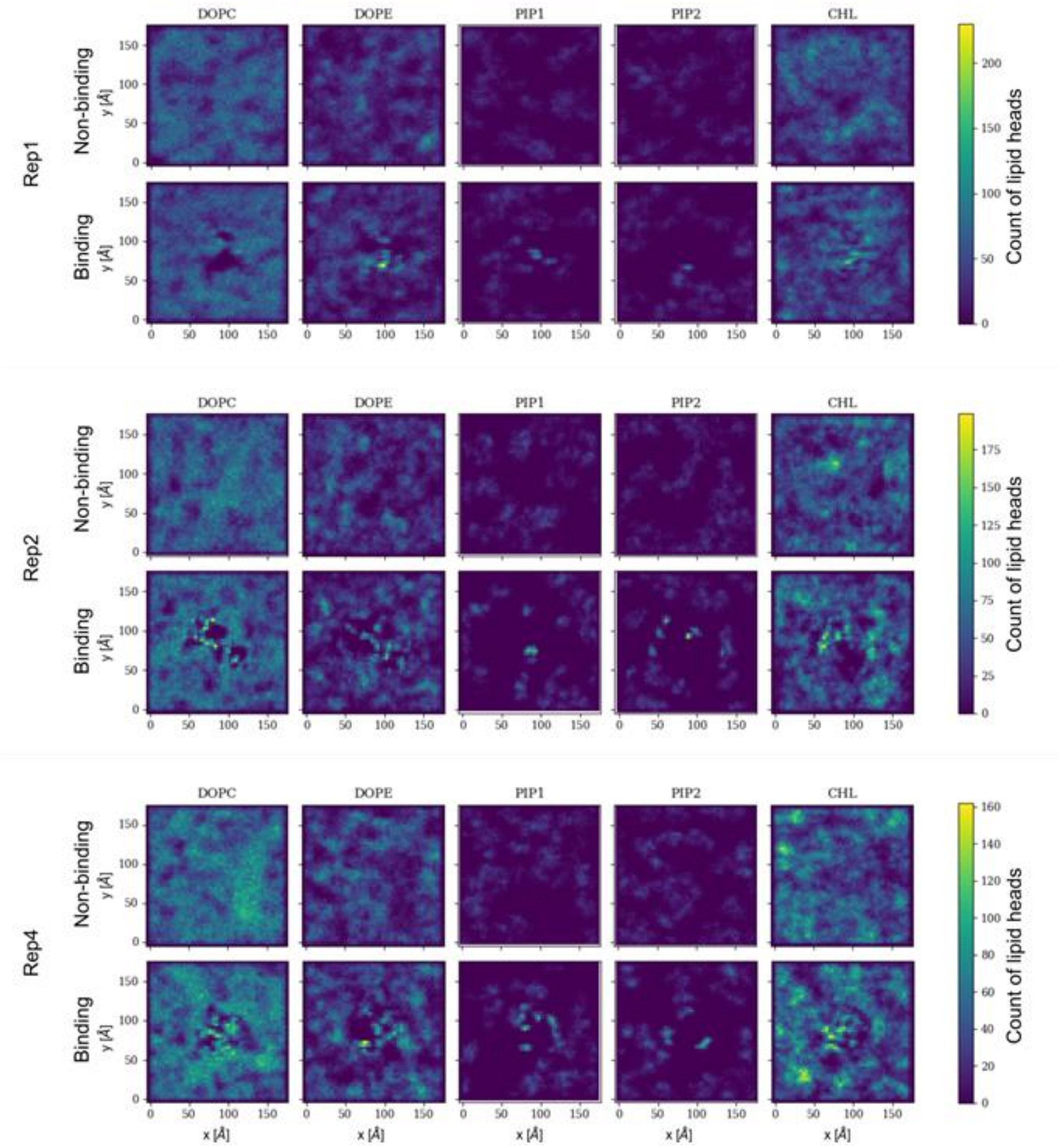

Figure S8. 2D maps for cumulative lipid sorting in Rep1 (top row), Rep2 (middle row), and Rep4 (bottom row). The color bar is the cumulative number of lipids in each square of the 2D histogram. Lipids were represented by either the phosphorus atom (P) for PC, PE, and inositol species, or the oxygen atom in the hydroxyl group (O3) in cholesterol over the last 500ns of the trajectory. Color intensity changes from dark blue to bright yellow as the concentration of lipid increases in the respective area.

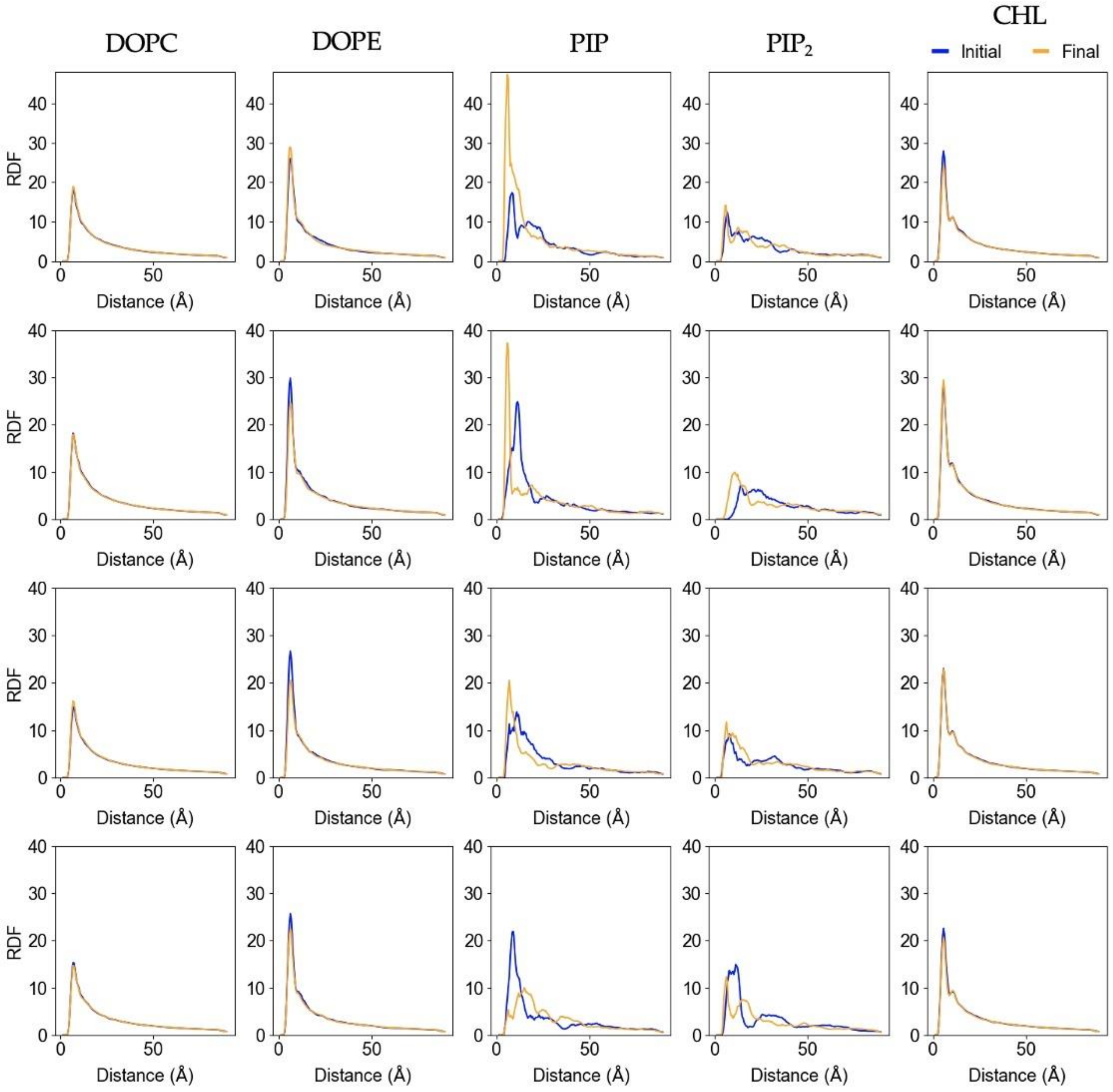

Figure S9. Lipid-lipid RDFs for each species in the binding leaflet upon initial contact vs at the end of the trajectory shown in blue and orange, respectively. Phosphate atoms (P) were used for the analysis of PC, PE, and inositol lipids, whereas the oxygen atom (O3) of the hydroxyl group in cholesterol (O3) were selected as representative atoms for this analysis. Initial contact analysis was performed over 50ns, the values corresponding to the end of the trajectory were averaged over the last 100ns. Each row corresponds to Rep1, Rep2, Rep3, and Rep4, respectively. The columns are labeled with the corresponding lipid species.

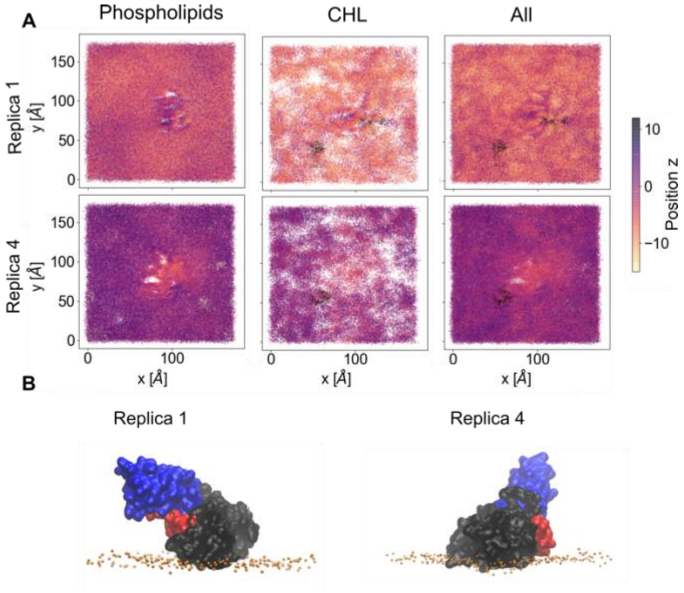

Figure S10. a. Cumulative plots for the relative position of phosphate atoms (left), hydroxyl oxygen from cholesterol (middle), and all lipids (right). The color bar indicates height with respect to the initial position of the respective atoms in the analysis period (i.e., the first frame of the last 500ns of trajectory). Color intensity changes from pale yellow to dark purple as the z-position of atoms increases; white patches indicate absence of the corresponding lipid atoms. **b.** Snapshots of the bound protein to the membrane for Rep1 and Rep4; the orange spheres show the relative position of the phosphate group of lipids in the binding leaflet. The 4HB is shown in blue, brace in red, and the PsK in black. The panel for *all lipids* for Rep4 shows a small incision on the membrane surface caused by the insertion of the protein.
